# Supplementary material for: Single-cell RNA-sequencing analysis of the developing mouse inner ear identifies molecular logic of auditory neuron diversification
Source: Nat Commun. 2022 Jul 5;13:3878. doi: 10.1038/s41467-022-31580-1 (PMC9256748; doi:10.1038/s41467-022-31580-1)
Supplement: Supplementary file 1 — Supplementary Information [file 41467_2022_31580_MOESM1_ESM.pdf]

Single cell RNAseq analysis of the developing mouse inner ear identifies molecular logic of auditory neuron diversification before birth

Charles Petitpré*/Louis Faure*, Phoebe Uhl, Paula Fontanet, Iva Filova, Gabriela Pavlinkova, Igor Adameyko, Saida Hadjab, & Francois Lallemend

Supplementary figures 1-9

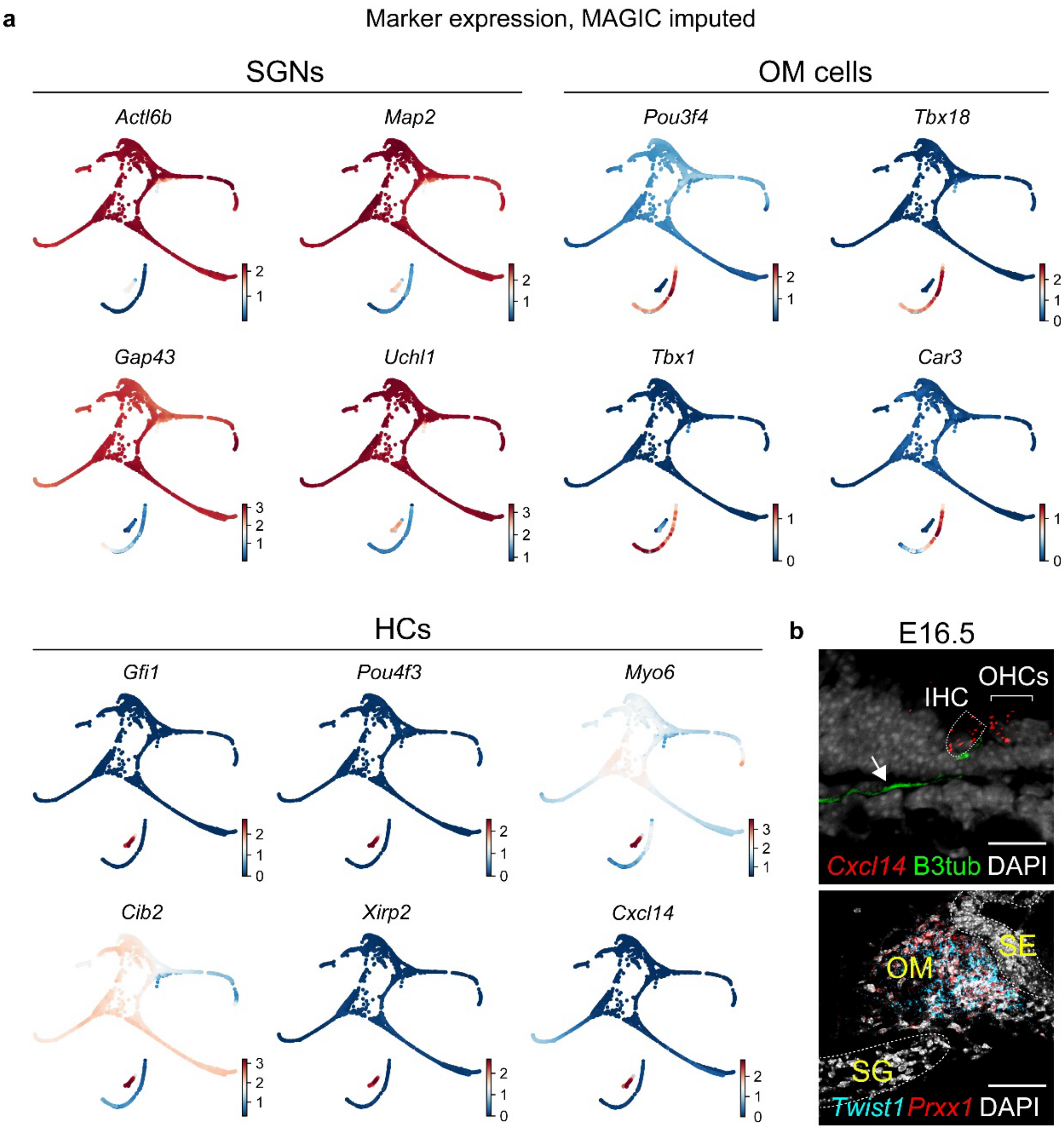

Supplementary Fig. 1 Marker expression in our scRNAseq dataset. a Plots showing expression of marker genes for SGNs, HCs and OM cells. **b** In vivo confirmation of marker genes for HCs (top panel) and OM cells (bottom panel) using immunostaining and RNAscope on cochlea sections from E16.5 WT mice. *Cxcl14* marks HCs innervated by βIII-tubulin^{+} spiral ganglion afferents (arrow in top panel). *Twist1* and *Prrx1* mark OM cells. Note that during the RNAscope procedure, at this early stage, some cells of the OM population can be washed out, which can result in a lower number of OM cells around the spiral ganglion. OM: otic mesenchyme; SE: sensory epithelium: SG: spiral ganglion.

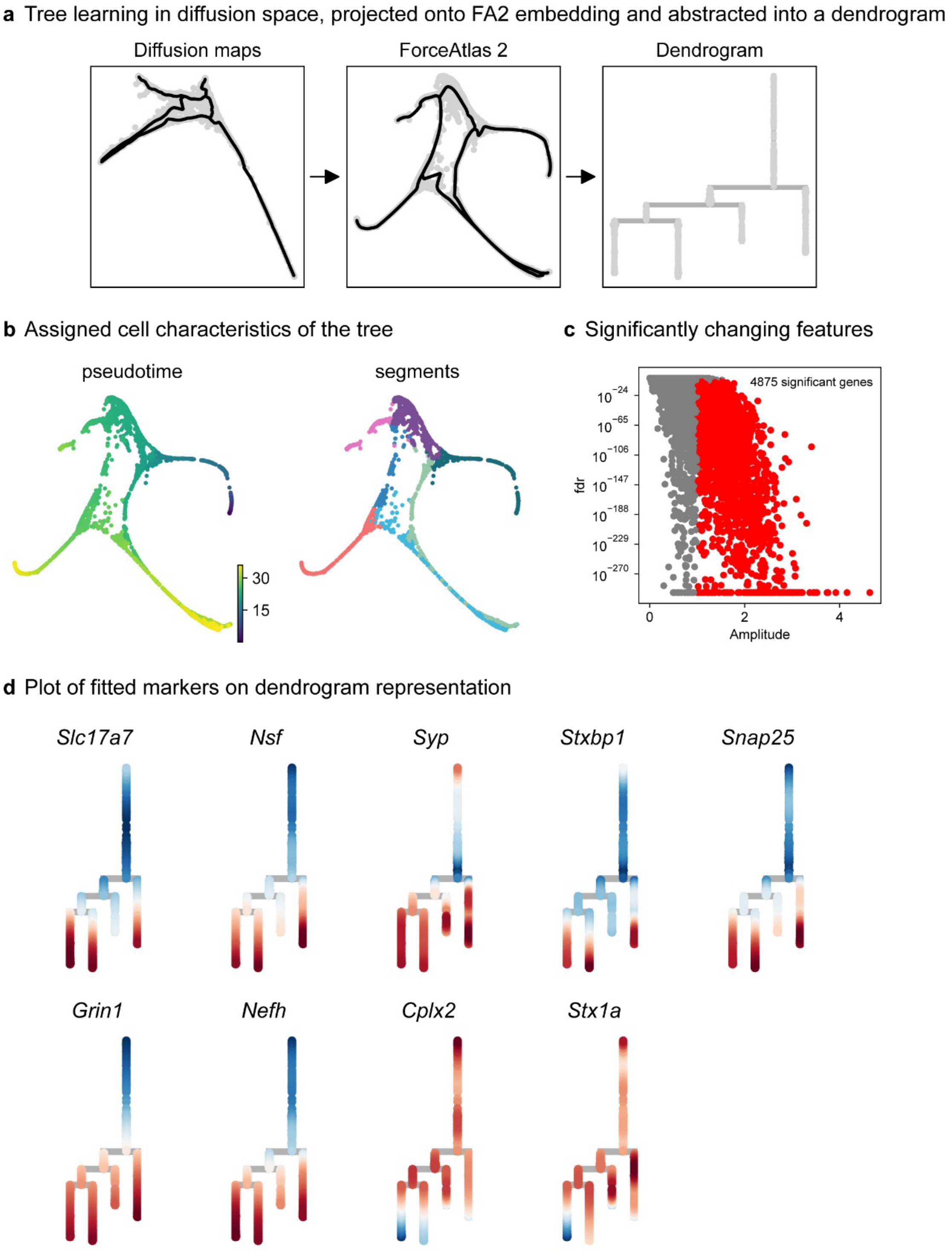

Supplementary Fig. 2 Characterization of branching tree of developing SGNs. a Steps of the force-directed layout embedding representation of the developmental branching tree of SGNs and of its second representation in a dendrogram, showing the initial part of the tree on top. **b** Cells are colored according to their pseudotime value (left) or cell lineage trajectory (right), on the branching tree. **c** Number of changing features (or unique transcripts) along the differentiation tree. **d** Dendrograms showing expression of genes associated with neuronal maturation/function.

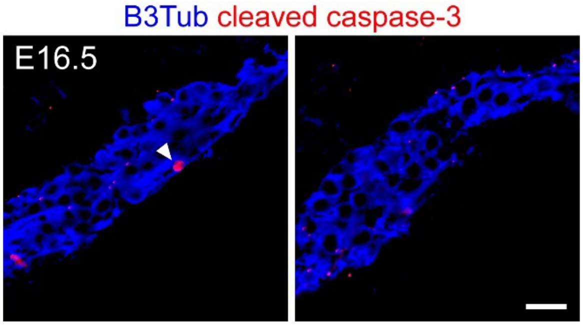

Supplementary Fig. 3 Limited apoptosis in SGNs of 16.5 *Neurod1^{cKO}* mice. Two illustrative microphotographs of an immunostaining for betaIII-tubulin and cleaved caspase-3 on SGN sections of E16.5 *Neurod1^{cKO}* mice. Note the presence of cleaved caspase-3 in one neuron in the left panel. Scale bar: 20 µm.

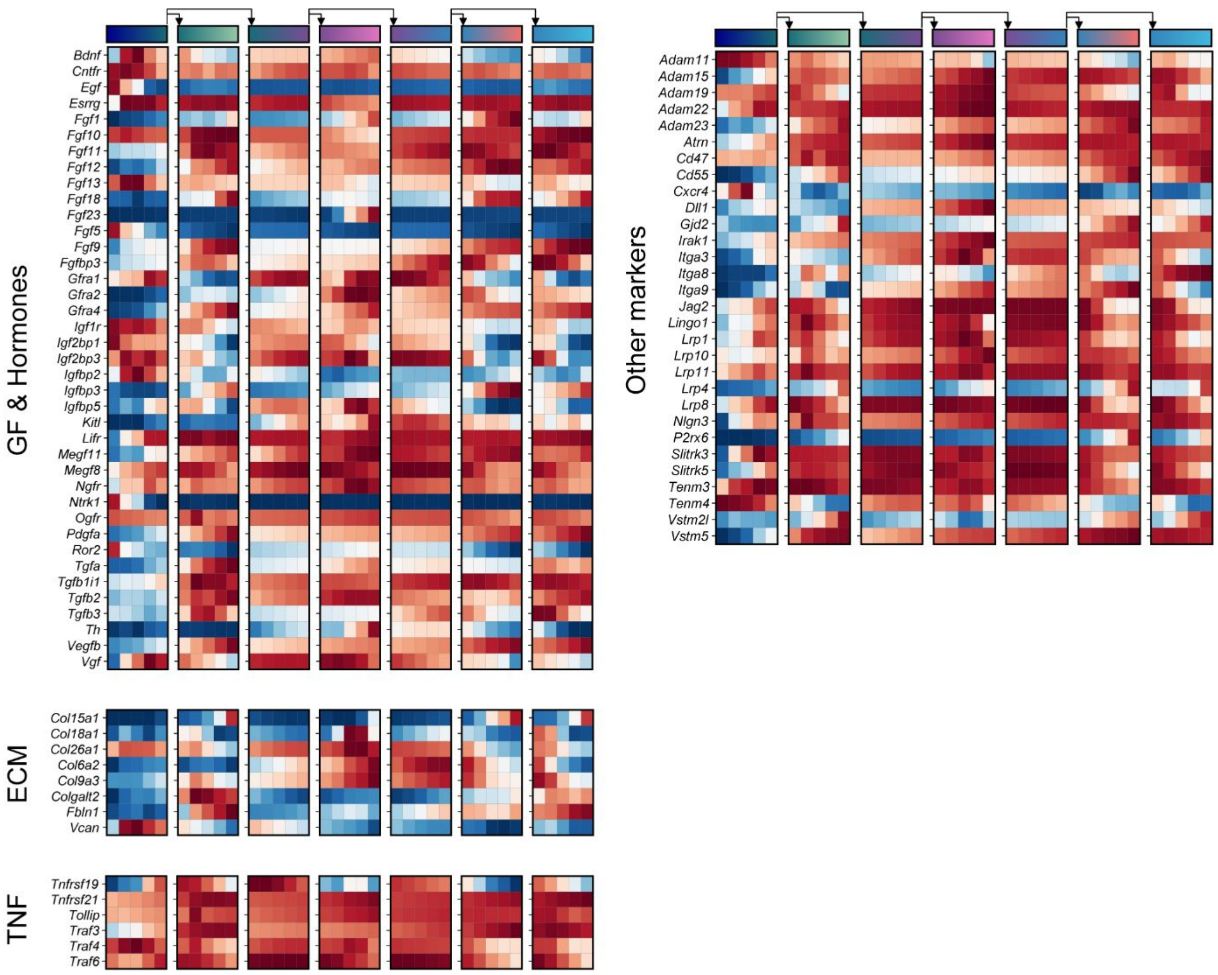

Supplementary Fig. 4 Cell-cell communication signature of differentiating SGNs, organized by gene names. Expression of genes linked to growth factor (GF) and hormone signaling, extracellular matrix (ECM), tumor necrosis factor (TNF) pathway and other marker genes in each neuronal trajectory. The color bars at the top indicate cell states as in Fig. 2a.

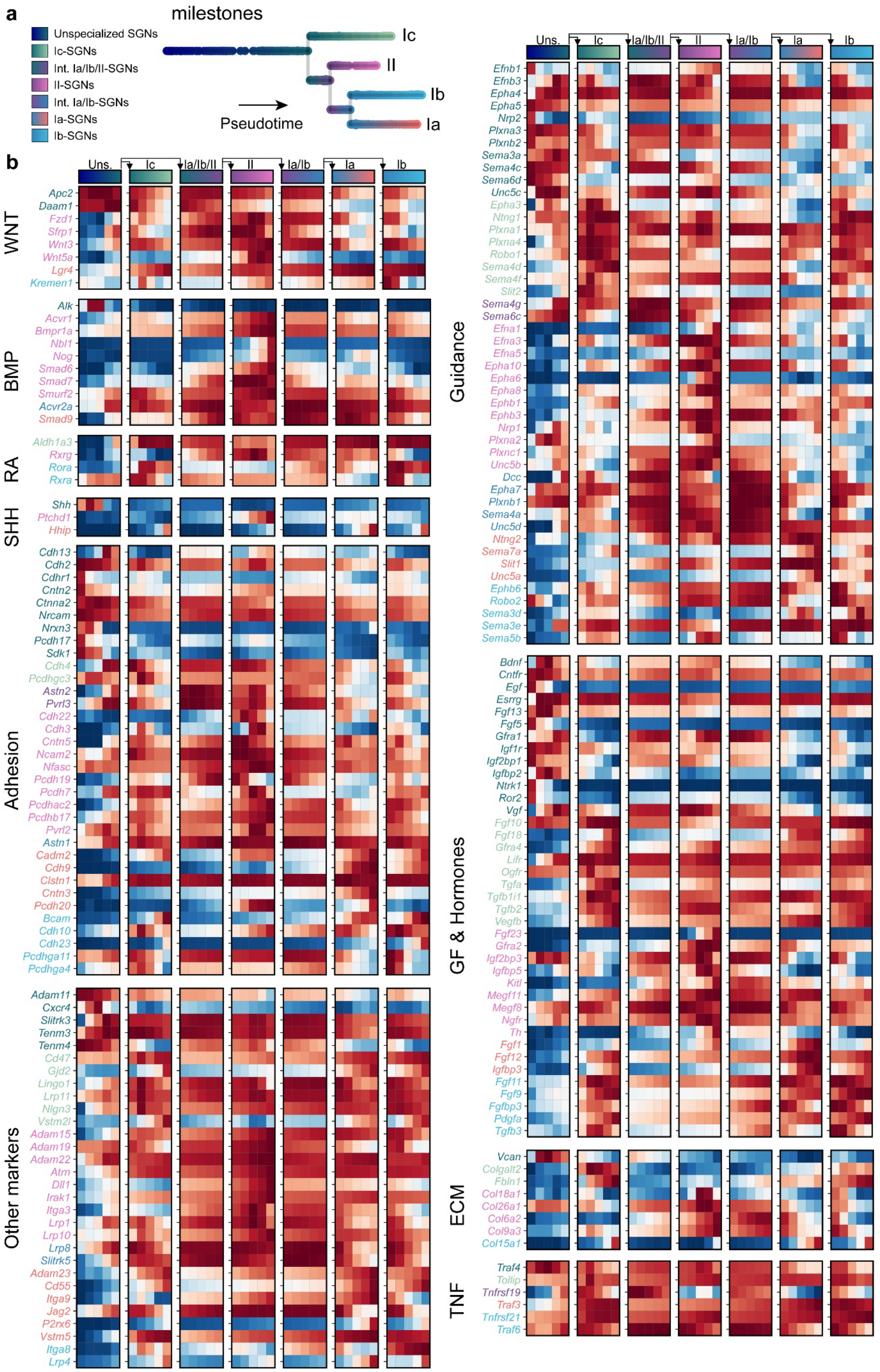
a
milestones
Unspecialized SGNs
Ic-SGNs
Int. Ia/Ib/II-SGNs
II-SGNs
Int. Ia/Ib-SGNs
Ia-SGNs
Ib-SGNs
Ic
II
Ib
Ia
Pseudotime
b
Uns.
Ic
Ia/Ib/II
II
Ia/Ib
Ia
Ib
WNT
Apc2
Daam1
Fzd1
Sfrp1
Wnt3
Wnt5a
Lgr4
Kremen1
BMP
Alk
Acvr1
Bmpr1a
Nbl1
Nog
Smad6
Smad7
Smurf2
Acvr2a
Smad9
RA
Aldh1a3
Rxrg
Rora
Rxra
SHH
Shh
Ptchd1
Hhip
Adhesion
Cdh13
Cdh2
Cdhr1
Cntn2
Ctnna2
Nrcam
Nrxn3
Pcdh17
Sdk1
Cdh4
Pcdhgc3
Astn2
Pvrl3
Cdh22
Cdh3
Cntn5
Ncam2
Nfasc
Pcdh19
Pcdh7
Pcdhac2
Pcdhb17
Pvrl2
Astn1
Cadm2
Cdh9
Clstn1
Cntn3
Pcdh20
Bcam
Cdh10
Cdh23
Pcdhga11
Pcdhga4
Other markers
Adam11
Cxcr4
Slitrk3
Tenm3
Tenm4
Cd47
Gjd2
Lingo1
Lrp11
Nlgn3
Vstm2l
Adam15
Adam19
Adam22
Atrn
Dll1
Irak1
Itga3
Lrp1
Lrp10
Lrp8
Slitrk5
Adam23
Cd55
Itga9
Jag2
P2rx6
Vstm5
Itga8
Lrp4
Guidance
Efnb1
Efnb3
Epha4
Epha5
Nrp2
Plxna3
Plxnb2
Sema3a
Sema4c
Sema6d
Unc5c
Epha3
Ntng1
Plxna1
Plxna4
Robo1
Sema4d
Sema4f
Slit2
Sema4g
Sema6c
Efna1
Efna3
Efna5
Epha10
Epha6
Epha8
Ephb1
Ephb3
Nrp1
Plxna2
Plxnc1
Unc5b
Dcc
Epha7
Plxnb1
Sema4a
Unc5d
Ntng2
Sema7a
Slit1
Unc5a
Ephb6
Robo2
Sema3d
Sema3e
Sema5b
GF & Hormones
Bdnf
Cntfr
Egf
Esrrg
Fgf13
Fgf5
Gfra1
Igf1r
Igf2bp1
Igfbp2
Ntrk1
Ror2
Vgf
Fgf10
Fgf18
Gfra4
Lifr
Ogfr
Tgfa
Tgfb1i1
Tgfb2
Vegfb
Fgf23
Gfra2
Igf2bp3
Igfbp5
Kitl
Megf11
Megf8
Ngfr
Th
Fgf1
Fgf12
Igfbp3
Fgf11
Fgf9
Fgfbp3
Pdgfa
Tgfb3
ECM
Vcan
Colgalt2
Fbln1
Col18a1
Col26a1
Col6a2
Col9a3
Col15a1
TNF
Traf4
Tollip
Tnfrsf19
Traf3
Tnfrsf21
Traf6

Supplementary Fig. 5 Cell-cell communication signatures defining SGN differentiation, organized by neuronal trajectories. **a** Dendrogram recapitulating the branched trajectory of the developing SGNs based on the transcriptional similarity of pseudotime-ordered cells. **b** Expression of genes linked to morphogen signaling, axon guidance, cell adhesion, growth factor (GF) and hormone signaling, extracellular matrix (ECM), tumor necrosis factor (TNF) pathway and other marker genes in each neuronal trajectory. The color bars at the top and color codes for the genes indicate cell states as in **a** and in Fig. 2a.

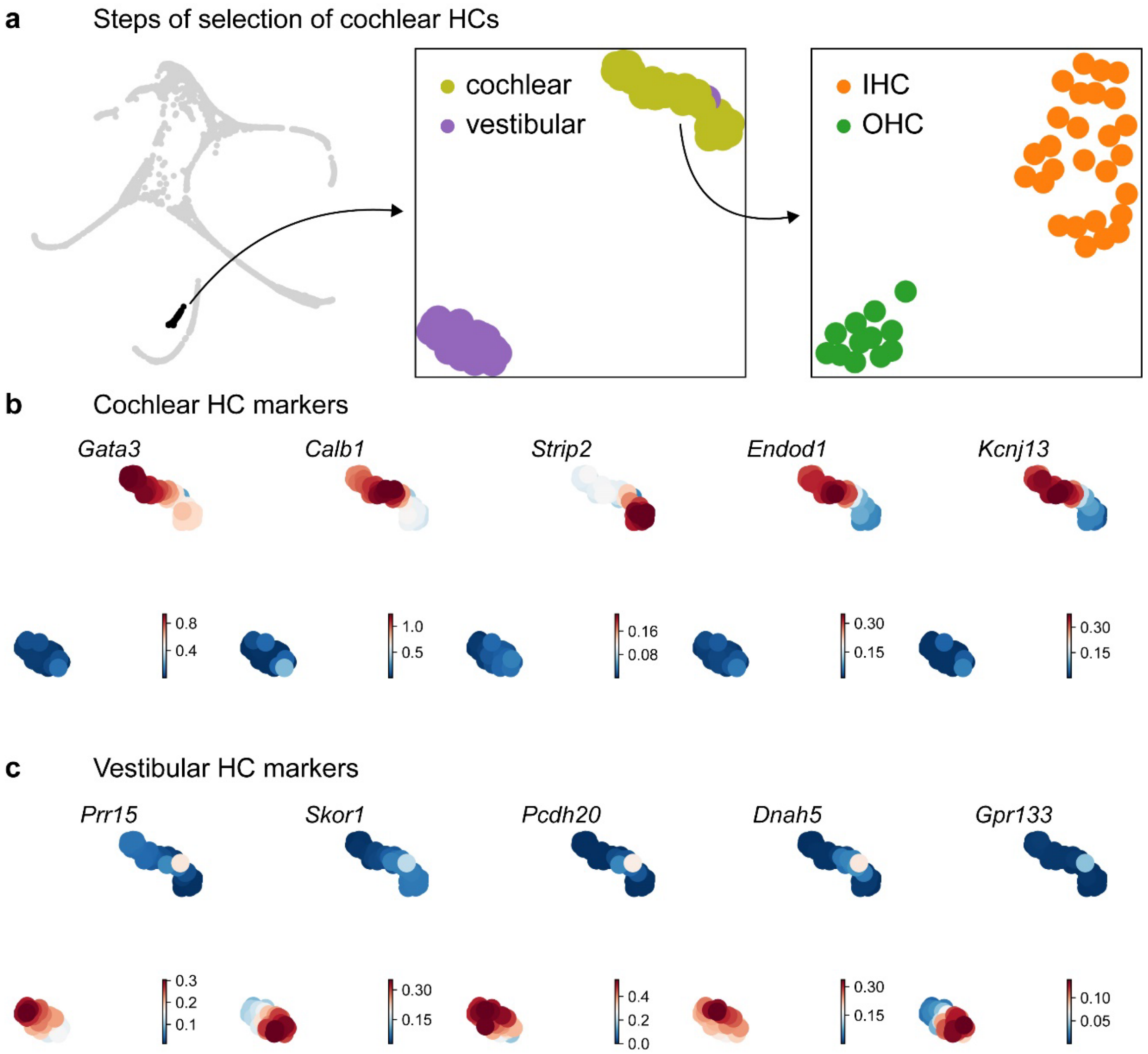

Supplementary Fig. 6 Selection of cochlear hair cells. a Identification of cochlear versus vestibular HCs (VHCs) from the FA plot (from fig. 1a). The center UMAP represents cochlear versus vestibular HCs based on gene scoring of the genes shown on b and c. A second UMAP identifies IHC versus OHCs, as detailed in Fig. 5. **b**, **c** UMAP of the vestibular and cochlear HCs showing specific marker genes expressed in either cochlear or vestibular HCs (Scheffer et al., 2015).

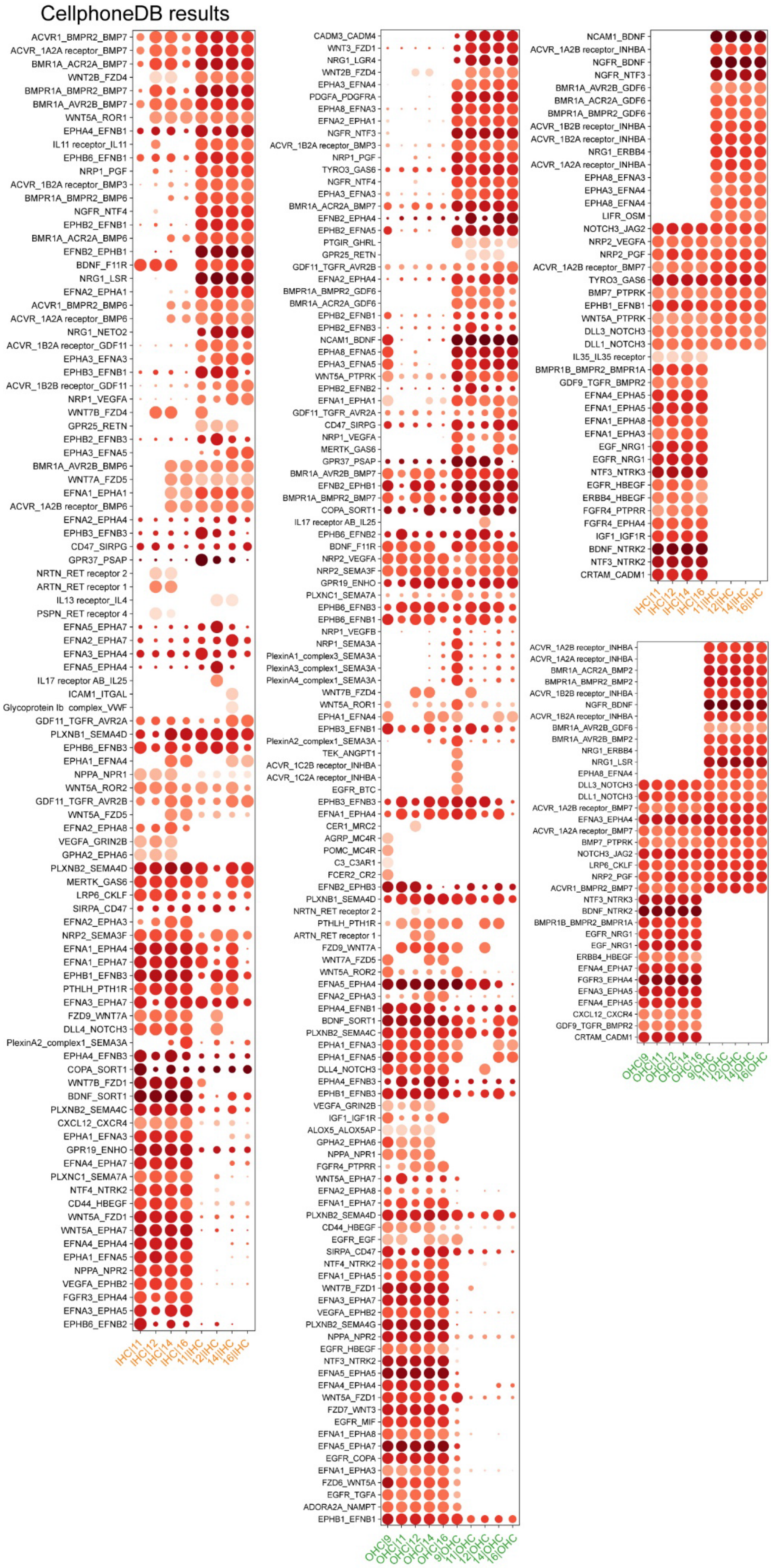
CellphoneDB results
ACVR1_BMPR2_BMP7
ACVR_1A2A receptor_BMP7
BMR1A_ACR2A_BMP7
WNT2B_FZD4
BMPR1A_BMPR2_BMP7
BMR1A_AVR2B_BMP7
WNT5A_ROR1
EPHA4_EFNB1
IL11 receptor_IL11
EPHB6_EFNB1
NRP1_PGF
ACVR_1B2A receptor_BMP3
BMPR1A_BMPR2_BMP6
NGFR_NTF4
EPHB2_EFNB1
BMR1A_ACR2A_BMP6
EFNB2_EPHB1
BDNF_F11R
NRG1_LSR
EFNA2_EPHA1
ACVR1_BMPR2_BMP6
ACVR_1A2A receptor_BMP6
NRG1_NETO2
ACVR_1B2A receptor_GDF11
EPHA3_EFNA3
EPHB3_EFNB1
ACVR_1B2B receptor_GDF11
NRP1_VEGFA
WNT7B_FZD4
GPR25_RETN
EPHB2_EFNB3
EPHA3_EFNA5
BMR1A_AVR2B_BMP6
WNT7A_FZD5
EFNA1_EPHA1
ACVR_1A2B receptor_BMP6
EFNA2_EPHA4
EPHB3_EFNB3
CD47_SIRPG
GPR37_PSAP
NRTN_RET receptor 2
ARTN_RET receptor 1
IL13 receptor_IL4
PSPN_RET receptor 4
EFNA5_EPHA7
EFNA2_EPHA7
EFNA3_EPHA4
EFNA5_EPHA4
IL17 receptor AB_IL25
ICAM1_ITGAL
Glycoprotein Ib complex_VWF
GDF11_TGFR_AVR2A
PLXNB1_SEMA4D
EPHB6_EFNB3
EPHA1_EFNA4
NPPA_NPR1
WNT5A_ROR2
GDF11_TGFR_AVR2B
WNT5A_FZD5
EFNA2_EPHA8
VEGFA_GRIN2B
GPHA2_EPHA6
PLXNB2_SEMA4D
MERTK_GAS6
LRP6_CKLF
SIRPA_CD47
EFNA2_EPHA3
NRP2_SEMA3F
EFNA1_EPHA4
EFNA1_EPHA7
EPHB1_EFNB3
PTHLH_PTH1R
EFNA3_EPHA7
FZD9_WNT7A
DLL4_NOTCH3
PlexinA2_complex1_SEMA3A
EPHA4_EFNB3
COPA_SORT1
WNT7B_FZD1
BDNF_SORT1
PLXNB2_SEMA4C
CXCL12_CXCR4
EPHA1_EFNA3
GPR19_ENHO
EFNA4_EPHA7
PLXNC1_SEMA7A
NTF4_NTRK2
CD44_HBEGF
WNT5A_FZD1
WNT5A_EPHA7
EFNA4_EPHA4
EPHA1_EFNA5
NPPA_NPR2
VEGFA_EPHB2
FGFR3_EPHA4
EFNA3_EPHA5
EPHB6_EFNB2
IHC|11
IHC|12
IHC|14
IHC|16
11|IHC
12|IHC
14|IHC
16|IHC
CADM3_CADM4
WNT3_FZD1
NRG1_LGR4
WNT2B_FZD4
EPHA3_EFNA4
PDGFA_PDGFRA
EPHA8_EFNA3
EFNA2_EPHA1
NGFR_NTF3
ACVR_1B2A receptor_BMP3
NRP1_PGF
TYRO3_GAS6
NGFR_NTF4
EPHA3_EFNA3
BMR1A_ACR2A_BMP7
EFNB2_EPHA4
EPHB2_EFNA5
PTGIR_GHRL
GPR25_RETN
GDF11_TGFR_AVR2B
EFNA2_EPHA4
BMPR1A_BMPR2_GDF6
BMR1A_ACR2A_GDF6
EPHB2_EFNB1
EPHB2_EFNB3
NCAM1_BDNF
EPHA8_EFNA5
EPHA3_EFNA5
WNT5A_PTPRK
EPHB2_EFNB2
EFNA1_EPHA1
GDF11_TGFR_AVR2A
CD47_SIRPG
NRP1_VEGFA
MERTK_GAS6
GPR37_PSAP
BMR1A_AVR2B_BMP7
EFNB2_EPHB1
BMPR1A_BMPR2_BMP7
COPA_SORT1
IL17 receptor AB_IL25
EPHB6_EFNB2
BDNF_F11R
NRP2_VEGFA
NRP2_SEMA3F
GPR19_ENHO
PLXNC1_SEMA7A
EPHB6_EFNB3
EPHB6_EFNB1
NRP1_VEGFB
NRP1_SEMA3A
PlexinA1_complex3_SEMA3A
PlexinA3_complex1_SEMA3A
PlexinA4_complex1_SEMA3A
WNT7B_FZD4
WNT5A_ROR1
EPHA1_EFNA4
EPHB3_EFNB1
PlexinA2_complex1_SEMA3A
TEK_ANGPT1
ACVR_1C2B receptor_INHBA
ACVR_1C2A receptor_INHBA
EGFR_BTC
EPHB3_EFNB3
EFNA1_EPHA4
CER1_MRC2
AGRP_MC4R
POMC_MC4R
C3_C3AR1
FCER2_CR2
EFNB2_EPHB3
PLXNB1_SEMA4D
NRTN_RET receptor 2
PTHLH_PTH1R
ARTN_RET receptor 1
FZD9_WNT7A
WNT7A_FZD5
WNT5A_ROR2
EFNA5_EPHA4
EFNA2_EPHA3
EPHA4_EFNB1
BDNF_SORT1
PLXNB2_SEMA4C
EPHA1_EFNA3
EPHA1_EFNA5
DLL4_NOTCH3
EPHA4_EFNB3
EPHB1_EFNB3
VEGFA_GRIN2B
IGF1_IGF1R
ALOX5_ALOX5AP
GPHA2_EPHA6
NPPA_NPR1
FGFR4_PTPRR
WNT5A_EPHA7
EFNA2_EPHA8
EFNA1_EPHA7
PLXNB2_SEMA4D
CD44_HBEGF
EGFR_EGF
SIRPA_CD47
NTF4_NTRK2
EFNA1_EPHA5
WNT7B_FZD1
EFNA3_EPHA7
VEGFA_EPHB2
PLXNB2_SEMA4G
NPPA_NPR2
EGFR_HBEGF
NTF3_NTRK2
EFNA5_EPHA5
EFNA4_EPHA4
WNT5A_FZD1
FZD7_WNT3
EGFR_MIF
EFNA1_EPHA8
EFNA5_EPHA7
EGFR_COPA
EFNA1_EPHA3
FZD6_WNT5A
EGFR_TGFA
ADORA2A_NAMPT
EPHB1_EFNB1
OHC|9
OHC|11
OHC|12
OHC|14
OHC|16
9|OHC
11|OHC
12|OHC
14|OHC
16|OHC
NCAM1_BDNF
ACVR_1A2B receptor_INHBA
NGFR_BDNF
NGFR_NTF3
BMR1A_AVR2B_GDF6
BMR1A_ACR2A_GDF6
BMPR1A_BMPR2_GDF6
ACVR_1B2B receptor_INHBA
ACVR_1B2A receptor_INHBA
NRG1_ERBB4
ACVR_1A2A receptor_INHBA
EPHA8_EFNA3
EPHA3_EFNA4
EPHA8_EFNA4
LIFR_OSM
NOTCH3_JAG2
NRP2_VEGFA
NRP2_PGF
ACVR_1A2B receptor_BMP7
TYRO3_GAS6
BMP7_PTPRK
EPHB1_EFNB1
WNT5A_PTPRK
DLL3_NOTCH3
DLL1_NOTCH3
IL35_IL35 receptor
BMPR1B_BMPR2_BMPR1A
GDF9_TGFR_BMPR2
EFNA4_EPHA5
EFNA1_EPHA5
EFNA1_EPHA8
EFNA1_EPHA3
EGF_NRG1
EGFR_NRG1
NTF3_NTRK3
EGFR_HBEGF
ERBB4_HBEGF
FGFR4_PTPRR
FGFR4_EPHA4
IGF1_IGF1R
BDNF_NTRK2
NTF3_NTRK2
CRTAM_CADM1
IHC|11
IHC|12
IHC|14
IHC|16
11|IHC
12|IHC
14|IHC
16|IHC
ACVR_1A2B receptor_INHBA
ACVR_1A2A receptor_INHBA
BMR1A_ACR2A_BMP2
BMPR1A_BMPR2_BMP2
ACVR_1B2B receptor_INHBA
NGFR_BDNF
ACVR_1B2A receptor_INHBA
BMR1A_AVR2B_GDF6
BMR1A_AVR2B_BMP2
NRG1_ERBB4
NRG1_LSR
EPHA8_EFNA4
DLL3_NOTCH3
DLL1_NOTCH3
ACVR_1A2B receptor_BMP7
EFNA3_EPHA4
ACVR_1A2A receptor_BMP7
BMP7_PTPRK
NOTCH3_JAG2
LRP6_CKLF
NRP2_PGF
ACVR1_BMPR2_BMP7
NTF3_NTRK3
BDNF_NTRK2
BMPR1B_BMPR2_BMPR1A
EGFR_NRG1
EGF_NRG1
ERBB4_HBEGF
EFNA4_EPHA7
FGFR3_EPHA4
EFNA3_EPHA5
EFNA4_EPHA5
CXCL12_CXCR4
GDF9_TGFR_BMPR2
CRTAM_CADM1
OHC|9
OHC|11
OHC|12
OHC|14
OHC|16
9|OHC
11|OHC
12|OHC
14|OHC
16|OHC

Supplementary Fig. 7 Cell-cell communication. CellPhoneDB analysis of the potential outgoing cell-to-cell signalling between HCs and SGNs. The two left plots show differentially (potentially) active signalling while the two plots on the right show those that are equally (potentially) active amongst pairs of cell types.

FACS reports

E14.5

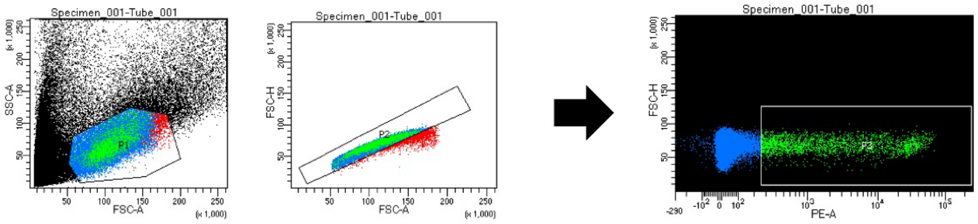

E15.5

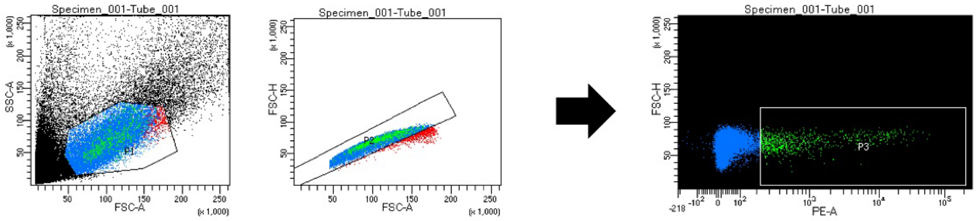

E16.5

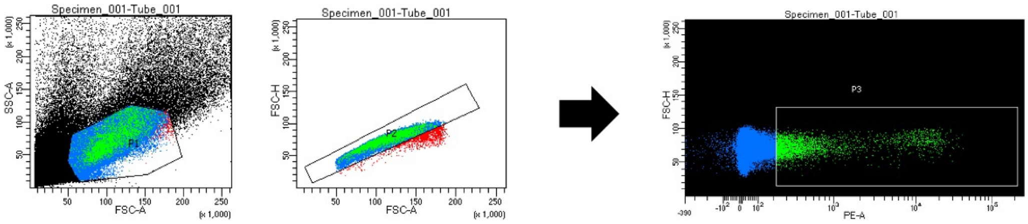

E17.5

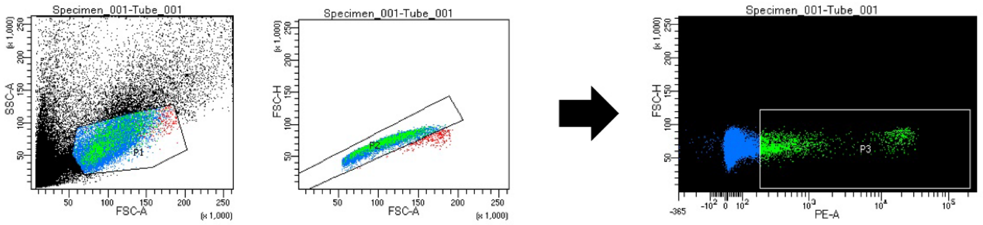

E18.5

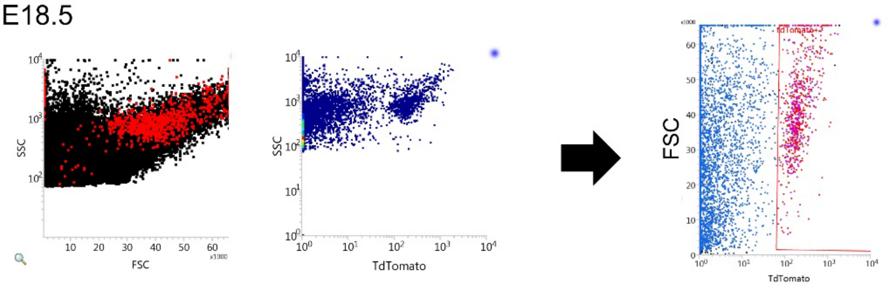

Supplementary Fig. 8 Illustrative graphs of the cell sorting of traced cells at the different time-points using flow cytometry. Note the different graph profiles at E18.5, which is due to a change of facility at the Karolinska institute. For each sorting, the first plot shows debris sorted out from the total events using FSC-A versus SSC-A plotting. Doublets were further gated out using FSC-A versus FSC-H plotting. Refinement of the single cells was performed by plotting FSC Area versus FSC. Finally, FSC-A versus PE-A (corresponding to TOMATO+ signal) to select cells that were TOMATO+.

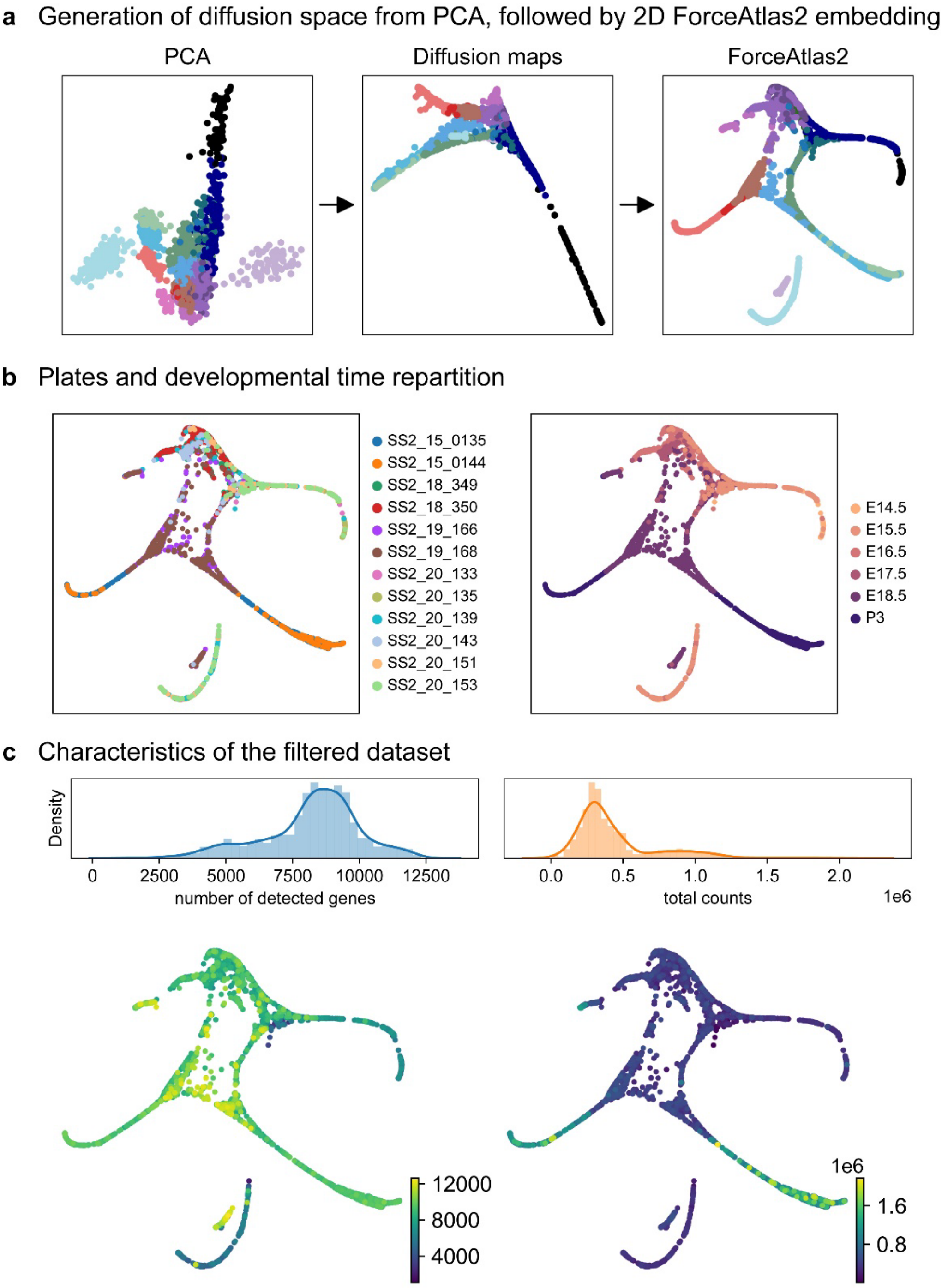

Supplementary Fig. 9 Quality control of the data and preprocessing. a Steps of generation from PCA (left) of the diffusion space (center) for trajectory analysis, and subsequently the Force Atlas2 2D embedding (right) for visualization. **b** Repartition of the extracted plates (left) and developmental times (right) on FA2 embedding. **c** Number of detected genes (left) and transcript (right).
