# Supplementary material for: Single-cell RNA-sequencing analysis of the developing mouse inner ear identifies molecular logic of auditory neuron diversification
Source: Nat Commun. 2022 Jul 5;13:3878. doi: 10.1038/s41467-022-31580-1 (PMC9256748; doi:10.1038/s41467-022-31580-1)
Supplement: Supplementary file 3 — Description of Additional Supplementary Files [file 41467_2022_31580_MOESM3_ESM.pdf]

**Title:** Supplementary Data 1
**Description:** related to Fig.1a_ leiden clusters_gene expression_all means

**Title:** Supplementary Data 2
**Description:** related to Fig. 1e_leiden clusters_all differentially expressed genes

**Title:** Supplementary Data 3
**Description:** related to Fig.2_ all differentially expressed genes along the differentiation tree

**Title:** Supplementary Data 4
**Description:** related to Fig.2_separate_paths

**Title:** Supplementary Data 5
**Description:** related to Fig.2h_original data

**Title:** Supplementary Data 6
**Description:** related to Fig.2i_original data

**Title:** Supplementary Data 7
**Description:** related to Fig.4_bifurcation analysis

**Title:** Supplementary Data 8
**Description:** related to Fig.5_vestibular hair cells

**Title:** Supplementary Data 9
**Description:** related to Fig.5_differentially expressed genes in IHCs and OHCs

**Title:** Supplementary Data 10
**Description:** related to Fig.6_deafness genes in IHCs and OHCs

**Title:** Supplementary Data 11
**Description:** related to Fig.6_deafness genes along SGN types trajectories

**Title:** Supplementary Data 12
**Description:** glial code genes
