# Supplementary material for: Single-cell RNA-sequencing analysis of the developing mouse inner ear identifies molecular logic of auditory neuron diversification
Source: Nat Commun. 2022 Jul 5;13:3878. doi: 10.1038/s41467-022-31580-1 (PMC9256748; doi:10.1038/s41467-022-31580-1)
Supplement: Supplementary file 16 — Reporting Summary [file 41467_2022_31580_MOESM16_ESM.pdf]

nature research

Corresponding author(s): Saida Hadjab & Francois Lallemend

Last updated by author(s): May 26, 2021

Reporting Summary

Nature Research wishes to improve the reproducibility of the work that we publish. This form provides structure for consistency and transparency in reporting. For further information on Nature Research policies, see our Editorial Policies and the Editorial Policy Checklist.

Statistics

For all statistical analyses, confirm that the following items are present in the figure legend, table legend, main text, or Methods section.

n/a	Confirmed	
☐	☒	The exact sample size (*n*) for each experimental group/condition, given as a discrete number and unit of measurement
☐	☒	A statement on whether measurements were taken from distinct samples or whether the same sample was measured repeatedly
☐	☒	The statistical test(s) used AND whether they are one- or two-sided<br>*Only common tests should be described solely by name; describe more complex techniques in the Methods section.*
☒	☐	A description of all covariates tested
☐	☒	A description of any assumptions or corrections, such as tests of normality and adjustment for multiple comparisons
☐	☒	A full description of the statistical parameters including central tendency (e.g. means) or other basic estimates (e.g. regression coefficient) AND variation (e.g. standard deviation) or associated estimates of uncertainty (e.g. confidence intervals)
☐	☒	For null hypothesis testing, the test statistic (e.g. *F*, *t*, *r*) with confidence intervals, effect sizes, degrees of freedom and *P* value noted<br>*Give P values as exact values whenever suitable.*
☒	☐	For Bayesian analysis, information on the choice of priors and Markov chain Monte Carlo settings
☒	☐	For hierarchical and complex designs, identification of the appropriate level for tests and full reporting of outcomes
☒	☐	Estimates of effect sizes (e.g. Cohen's *d*, Pearson's *r*), indicating how they were calculated

Our web collection on statistics for biologists contains articles on many of the points above.

Software and code

Policy information about availability of computer code

Data collection	single-cell data was collected by the Eukaryotic Single-cell Genomics facility at Science for Life Laboratory in Stockholm, Sweden. fastq demultiplexing was performed using deindexer (https://github.com/ws6/deindexer), aligment with STAR_2.4.2a(https://github.com/STAR) , deindexer commit version 19c200f186d8d2a77babe2435477998ec30e839e
Data analysis	Code for reproduction of analysis are deposited on the following github repository:<br>https://github.com/LouisFaure/sgnfates_paper<br>Raw data alignment and read counting were performed with STAR and featureCounts softwares. Main scRNAseq was performed with python packages scanpy, scFates, scvelo, cellrank

For manuscripts utilizing custom algorithms or software that are central to the research but not yet described in published literature, software must be made available to editors and reviewers. We strongly encourage code deposition in a community repository (e.g. GitHub). See the Nature Research guidelines for submitting code & software for further information.

Data

Policy information about availability of data

All manuscripts must include a data availability statement. This statement should provide the following information, where applicable:

- Accession codes, unique identifiers, or web links for publicly available datasets
- A list of figures that have associated raw data
- A description of any restrictions on data availability

Raw sequencing data are available on GEO database GSE165502, pagoda2 web application is also available on the Lallemend laboratory website (https://ki.se/en/neuro/lallemend-laboratory). Moreover, all the analyzed data are available by browsing and analysis via the gene Expression Analysis Resource (https://umgear.org/p?s=979d6f4b).

Tree fitting, pseudotime and bifurcation analysis was performed using scFates v0.4.0 python package, available via pypi: https://pypi.org/project/scFates/0.4.0/.
All codes and data for downstream analysis are deposited on the following github repo: https://github.com/LouisFaure/sgnfates_paper.
Figures with associated raw data: Fig. 1b-e; 2a-h; 3d,e; 4a-d,f; 5b,c.

Field-specific reporting

Please select the one below that is the best fit for your research. If you are not sure, read the appropriate sections before making your selection.

- [x] Life sciences
- [ ] Behavioural & social sciences
- [ ] Ecological, evolutionary & environmental sciences

For a reference copy of the document with all sections, see nature.com/documents/nr-reporting-summary-flat.pdf

Life sciences study design

All studies must disclose on these points even when the disclosure is negative.

Sample size	2274 cells sequenced after QC filtering. Initial filtering from sequenced plates to remove low quality cells or empty wells was done as followed: From the transcript count matrix, cells having less that 5x104 transcripts, less than 2500 genes and more than 25% of proportionof ERCC reads were filtered out.<br>No sample size calculation was performed, the number of filtered cell is sufficient because it covered all expected fates and cell types. This number is also sufficient given the high sensitivity of SmartSeq2 sequencing technique.
Data exclusions	glial code from glial contamination was filtered out, as described in the materials and methods section.<br>Glial code was removed in order to keep the contaminated SGN cells in further analysis, as these population were mirrors of clean SGN ones.
Replication	All experiment were done in replicate. For scRNAseq, Each plates sequenced is the result of the pooling of at least 3 embryos.<br>All replications were successful, no batch effect was detected among replicates of the same condition.
Randomization	No randomization was done as the only condition was wild type. The process of cell extraction of pooled embryos and sequencing is performed in an automated way, and does not discriminate a particular cell type.
Blinding	No blinding was done as mouse embryos were wild type. The process of cell extraction of pooled embryos and sequencing is performed in an automated way, and does not discriminate a particular cell type.<br>For experiments on mutant animals, blinding was not an option because the Neurod1 mutant cochlea already show visible phenotype on the total number of neurons, with fewer cells (as explained in the manuscript, with reference), therefore preventing the advantage of blinding.

Reporting for specific materials, systems and methods

We require information from authors about some types of materials, experimental systems and methods used in many studies. Here, indicate whether each material, system or method listed is relevant to your study. If you are not sure if a list item applies to your research, read the appropriate section before selecting a response.

Materials & experimental systems

n/a	Involved in the study	
[ ]	[x]	Antibodies
[x]	[ ]	Eukaryotic cell lines
[x]	[ ]	Palaeontology and archaeology
[ ]	[x]	Animals and other organisms
[x]	[ ]	Human research participants
[x]	[ ]	Clinical data
[x]	[ ]	Dual use research of concern

Methods

n/a	Involved in the study	
[x]	[ ]	ChIP-seq
[x]	[ ]	Flow cytometry
[x]	[ ]	MRI-based neuroimaging

Antibodies

Antibodies used	rabbit anti-calretinin (Swant) CR7697<br>rabbit anti-calbindin (Swant) CB-300<br>rabbit anti-RUNX1 (from Thomas Jessel lab)<br>mouse anti-betaIII-tubulin (Promega) G7121<br>rabbit anti-MyoVIIa (Abcam) ab3481<br>goat anti-peripherin (Everest Biotech) EB12405<br>rabbit anti-cleaved-caspase3 (Cell Signaling) #9661C<br>and DAPI (Invitrogen) D1306<br><br>Donkey anti rabbit 488 (Life Technologies) A-21206

	Donkey anti mouse 488 (Life Technologies) A-21202<br>Donkey anti goat 488 (Life Technologies) A-11055<br>Donkey anti rabbit 555 (Life Technologies) A-31572<br>Donkey anti mouse 555 (Life Technologies) A-31570<br>Donkey anti rabbit 647 (Life Technologies) A-31573<br>Donkey anti mouse 647 (Life Technologies) A-31571
Validation	anti-calretinin (https://www.swant.com/pdfs/Rabbit_anti_calretinin_7697.pdf)<br>anti-calbindin (https://www.swant.com/pdfs/Monoclonal_anti_calbindin_d28k_300.pdf)<br>anti-betaIII-tubulin (https://se.promega.com/products/protein-detection/primary-and-secondary-antibodies/anti_betaiii-tubulin-mab/?catNum=G7121)<br>anti-MyoVIIa (https://www.abcam.com/myosin-viiamyo7a-antibody-ab3481.html)<br>anti-peripherin (https://everestbiotech.com/product/goat-anti-peripherin-1-mouse-antibody/)<br>anti-cleaved-caspase3 (https://www.cellsignal.com/products/primary-antibodies/cleaved-caspase-3-asp175-antibody/9661)<br>anti-RUNX1 (https://elifesciences.org/articles/10874)

Animals and other organisms

Policy information about studies involving animals; ARRIVE guidelines recommended for reporting animal research

Laboratory animals	Animals of either sex were included in this study. Animals were group-housed, with food and water ad libitum, under 12 h light–dark cycle conditions, under standardized condition in the animal facility of Karolinska Institutet.
Wild animals	no wild animals were used in the study
Field-collected samples	no field collected samples were used in the study
Ethics oversight	All animal work was performed in accordance with the national guidelines and approved by the local ethics committee of Stockholm, Stockholms Norra djurförsöksetiska nämnd.

Note that full information on the approval of the study protocol must also be provided in the manuscript.
